# Genetic diversity of European maize landraces: Dataset on the molecular and phenotypic variation of derived doubled-haploid populations

**DOI:** 10.1016/j.dib.2022.108164

**Published:** 2022-04-12

**Authors:** Manfred Mayer, Armin C. Hölker, Thomas Presterl, Milena Ouzunova, Albrecht E. Melchinger, Chris-Carolin Schön

**Affiliations:** a Plant Breeding, TUM School of Life Sciences, Technical University of Munich, 85354 Freising, Germany; b KWS SAAT SE & Co. KGaA, 37574 Einbeck, Germany; c Institute of Plant Breeding, Seed Science and Population Genetics, University of Hohenheim, 70593 Stuttgart, Germany

**Keywords:** Plant genetic resources, Novel variation, Doubled-haploid lines, Quantitative traits, Early plant development, Field experiments, High resolution

## Abstract

Genetic variation is the basis of selection, evolution and breeding. Maize landraces represent a rich source of allelic diversity, but their efficient utilization in breeding and research has been hampered by their heterogeneous and heterozygous nature and insufficient information about most accessions. While molecular inventories of germplasm repositories are growing steadily, linking these data to meaningful phenotypes for quantitative traits is challenging.

Here, we present comprehensive molecular and phenotypic data for ∼1,000 doubled-haploid (DH) lines derived from three pre-selected European maize landraces. Due to their full homozygosity, the DH lines can be multiplied *ad libitum* and represent a powerful biological resource available to the community. The DH lines allow high-precision phenotyping in repeated experiments and reveal the full additive genetic variance of the population. The DH lines were evaluated for nine agronomically important, quantitative traits in multi-environment field trials comprising seven locations and two years. The DH populations revealed high genetic variance and high heritability for the analysed traits. The DH lines were genotyped with 600k SNP markers. After stringent quality filtering 500k markers remained for further analyses.

This is the largest resource of landrace derived DH material in maize, unprecedented in its structure and dimension. The presented data are ideal for linking molecular variation to meaningful phenotypes. They can be used for genome-wide association studies, genomic prediction, and population genetic analyses as well as for developing and testing statistical methods. All plant material is available to the community for conducting additional experiments, extending the panel of traits and environments, and for testing the landrace-derived lines in combination with other genetic material.

## Specifications Table

**Table:** 

Subject	Agronomy and Crop Science
Specific subject area	Plant genetics and breeding
Type of data	Tables
How the data were acquired	Genotypic data: DH lines were genotyped with the 600k Affymetrix® Axiom® Maize Array [1]; quality filtering and analyses were performed using R version 3.6.0 [2]; Missing values were imputed using Beagle version 5.0 [3]. Phenotypic data: Each DH line was replicated twice in each of the in total eleven environments (location-year combinations); experimental units were represented by rows of 20 plants; genotypes were randomly assigned to experimental units; traits were measured/scored manually; raw data and outliers were manually curated by inspection of residual plots; adjusted genotype means were calculated using R [2].
Data format	Raw Filtered Analysed
Description of data collection	Genotypic data: DNA was extracted from seedlings following Saghai-Maroof et al. [4]. The following traits were assessed in field trials: early vigour (at growth stages V4 and V6, whole plot, 1–9 score), early plant height (at V4 and V6, average over 3 plants, cm), final plant height (at R4, average over 3 plants, cm), days to male and female flowering (days until 50% of plants showed anthers/silks, d), lodging (at R6, whole plot, 1–9 score), tillering (at V9, whole plot, 1–9 score).
Data source location	Technical University of Munich, TUM School of Life Sciences, Plant Breeding, 85354 Freising, Germany Trial locations (country, latitude/longitude, years): • Einbeck (Germany, 51.81831/9.86674, 2017/18) • Roggenstein (Germany, 48.17985/11.32025, 2017/18) • Bernburg (Germany, 51.8246/11.70731, 2017) • Klein Wanzleben (Germany, 52.06887/11.36615, 2018) • Oberer Lindenhof (Germany, 48.47396/9.30498, 2017) • Golada (Spain, 42.77536/-8.10608, 2017/18) • Tomeza (Spain, 42.41072/-8.63441, 2017/18)
Data accessibility	Repository name: figshare Data identification number: 10.6084/m9.figshare.12137142.v1 Direct URL to data: https://doi.org/10.6084/m9.figshare.12137142.v1
Related research article	M. Mayer, A.C. Hölker, E. González-Segovia, E. Bauer, T. Presterl, M. Ouzunova, A.E. Melchinger, C.-C. Schön, Discovery of beneficial haplotypes for complex traits in maize landraces, Nat. Commun. 11 (2020) 4954. https://doi.org/10.1038/s41467-020-18683-3

## Value of the Data

• This is the largest resource of landrace derived DH-material in maize. It is unique in its structure and dimension.
• The biological material is available to the community and can serve as starting point for further research, extending the panel of traits and environments, testing the landrace-derived lines in combination with other genetic material and developing novel populations for (pre-)breeding and fine-mapping of candidate genes.
• The data presented here, together with the available underlying biological material and efficient analysis tools, open new avenues for the efficient utilization of the genetic diversity of maize landraces for enhancing elite germplasm with novel beneficial alleles.
• These data will benefit geneticists studying the genetic architecture of complex traits, gene bank curators aiming at making their inventories accessible for crop improvement, and breeders interested in harnessing native diversity for improving their breeding germplasm.
• These data are ideal for developing and testing novel statistical methods for genome-wide association studies, genomic prediction, haplotype construction, and population genetic analyses.
• The data can further be used to gain insights into maize history and the evolutionary consequences of maize expansion, environmental adaptation and breeding.

## 1 Data Description

This article presents high-dimensional genotypic and phenotypic data of three pre-selected European flint maize (*Zea mays* L. *ssp. mays*) landraces: Kemater Landmais Gelb (KE), Petkuser Ferdinand Rot (PE), and Lalin (LL). In total, 1,015 DH lines were derived from the three landraces (516 KE, 432 PE, and 67 LL). The DH lines represent samples of gametes segregating in the original landraces. Their full homozygosity allows *ad libitum* seed multiplication through selfing and therefore an evaluation in repeated experiments with any degree of precision desired. The DH lines reveal the full additive genetic variance of a population, without hidden allelic effects in heterozygous states. The three landraces can be assumed to be closest related to the European flint heterotic breeding pool, which therefore should be the target germplasm for introgression of novel beneficial alleles found in the DH populations. Accordingly, the hybrid performance of the DH lines should be evaluated in testcrosses with a complementary dent breeding line. All DH lines are available to the community through standard material transfer agreements upon request, no patents or plant breeders’ rights apply. The genotypes can be evaluated for additional traits for *per se* and testcross performance, expanding the dataset presented here according to particular research and breeding goals. The data and material presented here, support the research article “Discovery of beneficial haplotypes for complex traits in maize landraces” by Mayer et al. [5].

The DH lines were genotyped with the 600k Affymetrix® Axiom® Maize Array [1]. After stringent quality filtering 941 DH lines remained (501 KE, 409 PE, and 31 LL). The genotypic data provided consist of a raw data file (“Mayer_et_al_genotypes_DHlines_600k_raw.txt.gz”), comprising 941 DH lines and 616,201 markers, and a filtered and imputed data file, comprising 941 DH lines and 501,124 markers (“Mayer_et_al_genotypes_DHlines_600k_filteredImputed.txt.gz”). The raw and filtered data files contain 945 and 946 columns and 616,202 and 501,124 rows (header + individual markers), respectively. For both files, the first three columns contain the marker names and their physical position according to the B73 AGPv4 reference sequence [6]. Unmapped markers are indicated by the value '0′ for the chromosome. In the raw data file, column 4 indicates the marker quality class based on the genotype calling algorithm [1], while the remaining columns contain the individual genotype calls. Information on SNP IDs, genome positions according to B73 AGPv2 [7] (used for the array development), probe sets, and alleles are available at NCBI GEO as platform GPL18778 (http://www.ncbi.nlm.nih.gov/geo/query/acc.cgi?acc=GPL18778). The landrace membership of individual lines is indicated by the respective abbreviation (KE, PE, LL) included in the column headers. The filtered data file contains only markers with the best quality class, “Poly High Resolution” [1], and genotype scores coded as ‘0’ and ‘2’, without missing values. Columns 4 and 5 indicate the alleles corresponding to scores ‘0’ and ‘2’, respectively.

**Fig. 1:**
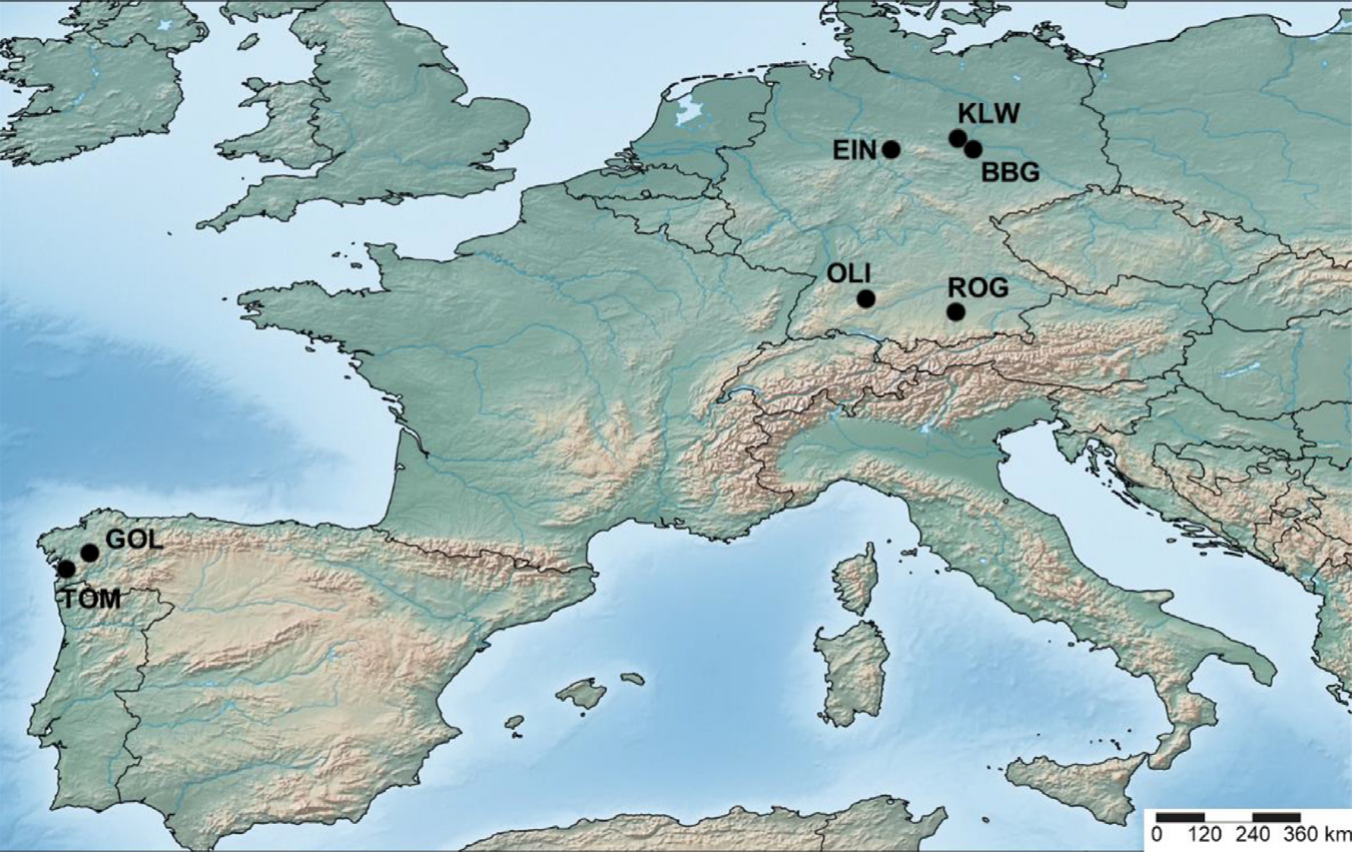
**Locations of the field trials conducted in 2017 and 2018**. Einbeck (EIN; 2017/18), Roggenstein (ROG; 2017/18), Bernburg (BBG; 2017), Klein Wanzleben (KLW; 2018), Oberer Lindenhof (OLI; 2017), Golada (GOL; 2017/18) and Tomeza (TOM; 2017/18).

The field experiments comprised 958 landrace-derived DH lines as well as 15 breeding lines and samples of the original landraces. Thereof, 899 DH lines (KE = 471, LL = 26, and PE = 402) passed the quality filtering based on genotypic data. The lines were evaluated for various traits in six and five locations in 2017 and 2018, respectively, resulting in up to eleven location-year combinations per DH line and trait (Fig. 1, Table 1). Total precipitation as well as average, minimum and maximum daily temperature during the vegetation period for each trial are described in Hölker et al. 2019 [8]. The nine traits for which data are provided are early vigour (EV; at growth stages V4 and V6, whole plot, 1–9 score), early plant height (PH; at V4 and V6, average over 3 plants, cm), final plant height (PH_final; at R4, average over 3 plants, cm), days to male (MF) and female (FF) flowering (days until 50% of plants showed anthers/silks, d), lodging (LO; at R6, whole plot, 1–9 score) and tillering (TILL; at V9, whole plot, 1–9 score). Heritabilities ranged between 0.86 and 0.96 [8], confirming high quality of the data. A detailed analysis of the phenotypic data, describing population means, genetic variances, genotype × environment interaction variances, as well as trait correlations, can be found in Hölker et al. 2019 [8]. Four phenotypic data files are provided here: the plot-level data of the 899 DH lines and checks (“Mayer_et_al_phenotypes_rawData.txt.gz”) including the plot number, location, year, environment, the genotype names, the design factors (lattice, replication, block) and the corresponding phenotypic values for the nine traits; a data file with best linear unbiased estimates (BLUEs) of genotypic values for each environment (“Mayer_et_al_phenotypes_BLUEs_perEnvironment.txt.gz”); a data file with BLUEs calculated across all environments (“Mayer_et_al_phenotypes_BLUEs_acrossAllEnvironments.txt.gz”); and a data file with BLUEs calculated across locations in 2017 (“Mayer_et_al_phenotypes_BLUEs_acrossSixLocations2017.txt.gz”).

**Table 1:** **Number of lines evaluated in each environment.** For each location-year combination, the number of entries evaluated, the number of DH lines after quality filtering and the number of checks (BL = breeding lines, LR = original landraces) are shown.

Location	Year	N entries	N lines after QC	N checks
Bernburg (BBG)	2017	1,000	461 KE 14 LL 393 PE	15 BL 3 LR

Einbeck (EIN)	2017	1,000	462 KE 14 LL 393 PE	15 BL 3 LR

	2018	800	365 KE 26 LL 365 PE	4 BL 3 LR

Golada (GOL)	2017	500	210 KE 7 LL 204 PE	15 BL 3 LR

	2018	500	222 KE 6 LL 240 PE	4 BL 3 LR

Klein Wanzleben (KLW)	2018	800	365 KE 26 LL 365 PE	4 BL 3 LR

Oberer Lindenhof (OLI)	2017	1,000	441 KE 13 LL 390 PE	15 BL 3 LR

Roggenstein (ROG)	2017	1,000	461 KE 14 LL 390 PE	15 BL 3 LR

	2018	800	365 KE 26 LL 365 PE	4 BL 3 LR

Tomeza (TOM)	2017	500	210 KE 7 LL 204 PE	15 BL 3 LR

	2018	500	222 KE 6 LL 240 PE	4 BL 3 LR

## 2 Experimental Design, Materials and Methods

### 2.1 Plant material

The flint maize landraces KE, LL and PE, originating from Austria, Spain, and Germany, respectively, were pre-selected from a broad panel of 35 European landraces based on population genetic analyses [9] and preliminary field trials evaluating the original landrace populations. Selection criteria were large molecular variation, limited population structure and low levels of linkage disequilibrium (LD) within the landraces, as well as large variation for early plant development and cold tolerance related traits. In total, 1,015 DH lines (516 KE, 432 PE, 67 LL) were derived from the three landraces using the in vivo haploid induction method [10]: pollinating plants of the original landraces with an inducer line, identifying haploid kernels with a colour marker, doubling chromosomes using colchicine, and selfing the resulting plants. The DH lines were multiplied for evaluation in repeated experiments. In addition, 15 breeding lines were evaluated as checks in the field trails: 14 flint lines, CH10 (Agroscope Changins-Wädenswil, Switzerland); D152, DK105, UH006, UH007, and UH009 (University of Hohenheim, Germany); EP1 and EP44 (Misión Biológica de Galicia, Consejo Superior de Investigaciones Científicas, CSIC, Spain); F03802, F2, F283, F64, and F7 (Institut National de la Recherche Agronomique, INRA, France); EC49A (Centro de Investigaciones Agrarias Mabegondo, Instituto Galego da Calidade Aumentaria, CIAM-INGACAL, Spain); and one dent line, F353 (INRA, France). The 14 flint breeding lines were selected to represent the genetic diversity of the European flint breeding germplasm. They were released between ∼1950 and 2010 and include very prominent lines (e.g. EP1, F2, F7, and DK105) that played a key role in European breeding programs. F353 represents an important line of the European dent breeding pool and was for example used as central line in the EU-NAM dent population [11]. F353 has also been used as tester in testcross evaluations of the landrace-derived DH lines [8], which will expand the dataset presented here for additional traits like silage yield.

### 2.2 Genotyping

**Fig. 2:**
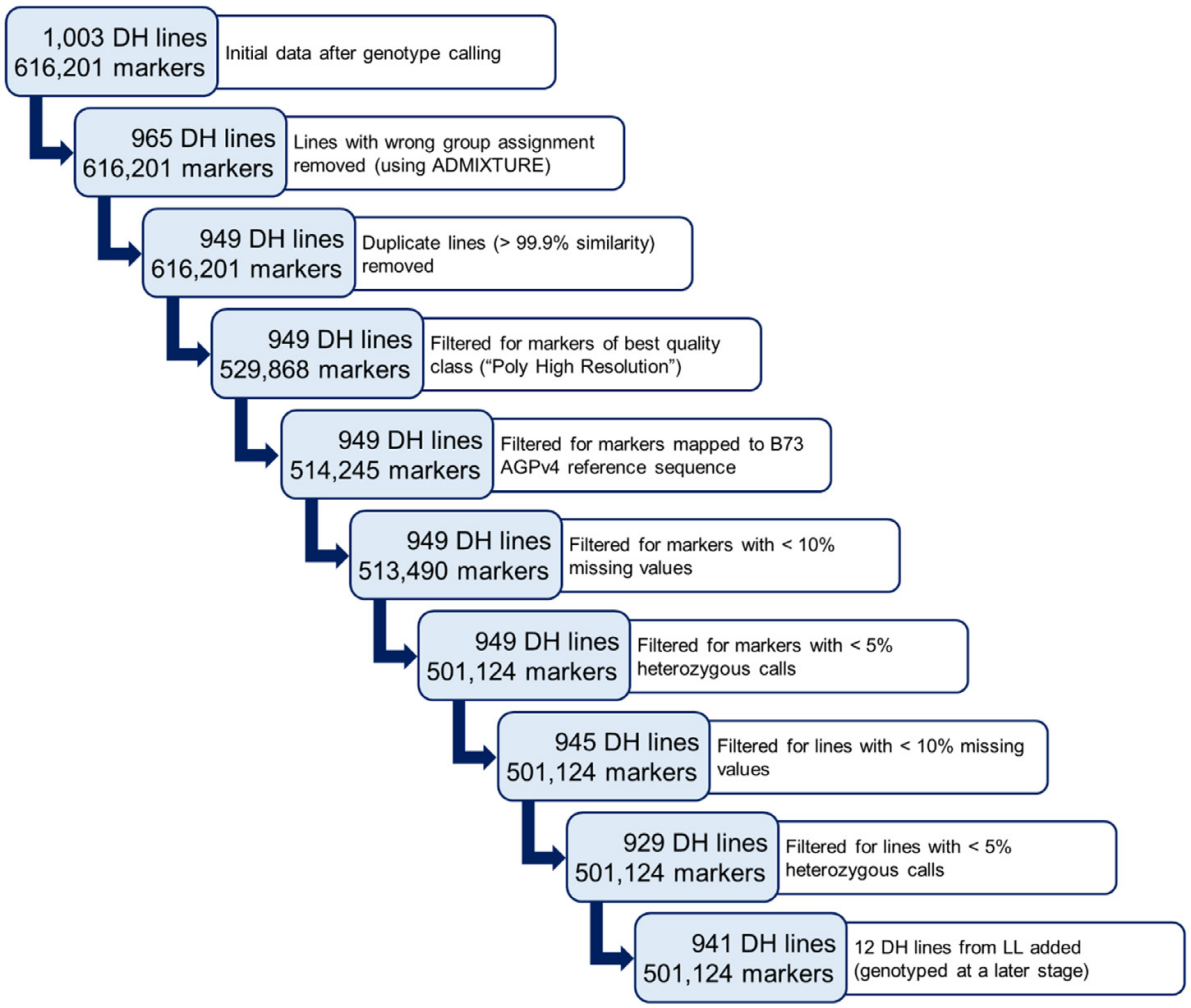
**Quality control (QC) for the 600k genotypic data**. Scheme shows the number of DH lines and markers remaining after each QC step. The final dataset of 941 DH lines and 501,124 markers was subsequently imputed and used for the analyses described in Mayer et al. [5].

DNA was extracted from leaf material of seedlings of each DH line following the protocol of Saghai-Maroof et al. [4] and each sample was processed on the Affymetrix GeneTitan® platform with the 600k Affymetrix® Axiom® Maize Array [1] following manufacturer's protocol. Raw hybridization intensity data processing, clustering, genotype calling, inclusion of inbreeding level information, off-target variant calling, and variant categorization according to genotype cluster metrics were performed according to the Axiom® Genotyping Solution Data Analysis Guide as described in Unterseer et al. [1]. A threshold of 0.90 for the variant call frequency instead of the default value (0.97) was applied. The initial genotype calling was performed for a combined set of 1,003 DH lines (516 KE, 432 PE, 55 LL) together with 2,150 other landrace-derived individuals, including e.g. samples from the original landraces [8,9]. The genotypic data of the 1,003 DH lines were extracted and the quality filtering steps as illustrated in Fig. 2 performed. In a later step, 600k genotyping data of 12 DH lines derived from landrace LL were added, resulting from a second round of DH production and separate genotype calling.

For each DH line, the proportion of ancestry attributable to each of the three landraces was estimated with the software ADMIXTURE [12] in a supervised mode, using genotyped plants of the original landraces as templates for pre-defining the groups KE, PE, and LL. DH lines with less than 75% concordance with the landrace to which they were assigned by pedigree records were removed as they might have been affected by cross-pollination. Duplicate lines, i.e. lines showing the same allele at > 99.9% of the markers, were excluded. Markers were filtered for the best quality class (“Poly High Resolution”) as defined by the calling algorithm [1]. Further, markers were filtered for a known physical position on the B73 AGPv4 reference sequence [6], < 10% missing values and < 5% heterozygous genotype calls. Subsequently, DH lines were filtered for < 10% missing values and < 5% heterozygous genotype calls. Finally, the genotypic data of 12 additional DH lines of LL were added, which met the same quality criteria. For the resulting dataset of 941 DH lines and 501,124 markers, remaining heterozygous genotype calls (0.19%) were set to missing as they can be assumed to represent technical artefacts, and all missing values were imputed separately for each landrace using Beagle version 5.0 [3]. The two datasets provided with the article both contain the filtered set of 941 DH lines but differ in the set of markers. The unimputed dataset contains the full set of 616,201 markers, whereas the filtered and imputed dataset with 501,124 markers was the basis for the analyses described in Mayer et al. [5].

### 2.3 Field experiments

The performance of the DH lines was evaluated in six and five locations in 2017 and 2018, respectively, resulting in eleven different environments (location-year combinations; Fig. 1, Table 1). In the German locations (BBG, EIN, OLI, ROG) 2017, 1,000 entries (958 DH lines plus checks) were evaluated using ten separate 10  ×  10 lattice designs with two replicates, including 14 flint and one dent breeding lines (duplicate entries) as well as the three original landraces (quadruple entries) as checks. In 2018, the number of lines tested was reduced, due to seed shortage and the exclusion of lines by the quality filtering based on the genotypic data, described above. As a result, 800 entries (756 DH lines plus checks) were tested in the German locations 2018, using eight 10  ×  10 lattice designs with two replicates, including three flint (DK105, EP1, F2) and one dent (F353) breeding lines as checks in each lattice and the three original landraces again as quadruple entries. In the Spanish locations (GOL, TOM), a randomly chosen subset of lines was evaluated in both years (500 entries: 458 and 468 DH lines plus checks in 2017 and 2018, respectively) using five 10  ×  10 lattice designs with two replicates. The inclusion of checks in the Spanish locations was analogous to the German locations in 2017 and 2018. In all trials, each entry referred to a single row plot with 3 m length and 20 kernels sown per plot. The distance between plots was 0.75 m, corresponding to a sowing density of about 8.9 kernels m^−2^.

Traits were measured/scored manually in the field. Experimental units were associated with plot numbers only and not with genotype names, i.e. investigators were blinded to group allocations during data collection. Early vigour was scored in growth stages V4 and V6 [13] on a 1-9 scale, assessing the visual appearance of a plot, taking into account the size (height, width, leaf area), the colour (greenness, discolorations) and health (necrosis, general appearance) of the plants, with the score of 1 representing very weak, small plants with a lot of discoloration and necrosis and 9 representing very vigorous, big, healthy looking plants without discolouration or necrosis. Early plant height was measured in stages V4 and V6 as the distance (in cm) from the soil surface to the highest tip of upwards stretched leaves. The plot value was calculated as the mean of three representative plants measured. Final Plant height was measured between stages R3 and R5 as the distance (in cm) from the soil surface to the lowest tassel branch, taking again the average of three representative plants measured per plot. Female and male flowering were defined as the number of days until 50% of the plants within a plot showed silks or shed pollen, respectively. Lodging was scored in stage R6 on a 1-9 scale, taking into account the number of affected plants as well as the severity of lodging (1 = no lodging, 9 = all plants show severe lodging). Tillering was scored between stages V8 and V10 on a 1-9 scale, taking into account the number of affected plants as well as the number and length of the tillers (1 = no tillers, 9 = all plants show many long tillers). Early vigour and plant height were assessed in all eleven environments. Female and male flowering were evaluated in ten and five environments, respectively, and lodging and tillering were scored in four and five environments, respectively.

For each trait, BLUEs of genotype means across environments were calculated using the model

$$y_{ijkop}=\mu +g_{i}+w_{j}+gw_{ij}+k_{k\left(j\right)}+r_{o\left(jk\right)}+b_{p\left(jko\right)}+e_{ijkop}$$

where $y_{ijkop}$ are the plot level observations; μ is the overall mean; $g_{i}$ is the effect of genotype *i*; $w_{j}$ is the effect of environment *j*; $gw_{ij}$ is the interaction effect for genotype *i* and environment *j*; $k_{k(j)}$ is the effect of the lattice *k* nested in environment *j*; $r_{o(jk)}$ is the effect of replicate *o* nested in lattice *k* and environment *j*; $b_{p(jko)}$ is the effect of block *p* nested in replicate *o*, lattice *k* and environment *j*; and $e_{ijkop}$ is the residual error. All effects except $g_{i}$ were treated as random. Residuals were assumed to be normally distributed with mean zero and two heterogeneous variances, one for DH lines and one for checks (breeding lines and original landraces). BLUEs of genotype means within each environment were calculated using the same model without environment-related model terms.

## CRediT authorship contribution statement

**Manfred Mayer:** Conceptualization, Investigation, Data curation, Formal analysis, Visualization, Writing – original draft. **Armin C. Hölker:** Investigation, Data curation, Formal analysis. **Thomas Presterl:** Investigation. **Milena Ouzunova:** Conceptualization, Funding acquisition, Investigation. **Albrecht E. Melchinger:** Funding acquisition, Investigation. **Chris-Carolin Schön:** Conceptualization, Funding acquisition, Investigation, Writing – review & editing.

## Declaration of Competing Interest

The authors declare that they have no known competing financial interests or personal relationships that could have appeared to influence the work reported in this paper.

## Data Availability

Data from Mayer et al. 2020 (Nat. Commun.) (Original data) (figshare).
